# Supplementary material for: Polyketide synthase gene clusters in the ericoid mycorrhizal fungus Oidiodendron maius: genome-wide prediction, expression profiling, and functional characterization of knockout mutants
Source: Front Plant Sci. 2026 Sep 16;17:1924430. doi: 10.3389/fpls.2026.1924430 (PMC13623903; doi:10.3389/fpls.2026.1924430)
Supplement: Supplementary file 1 [file SupplementaryFile1.zip › Supplementary Materials/Supplementary Folder 1.zip!/Oidma1_AssemblyScaffolds_Repeatmasked/knownclusterblast/cluster16/jgi.p_Oidma1_40831_mibig_hits.html]

| MIBiG Protein | Description | MIBiG Cluster | MiBiG Product | % ID | % Coverage | BLAST Score | E-value |
| --- | --- | --- | --- | --- | --- | --- | --- |
| EHA28232.1 | hypothetical\_protein | BGC0001143 | Azanigerone\_biosynthetic\_gene\_cluster | 49 | 100.0 | 301 | 1.4e-81 |
| XP\_011392665.1 | hypothetical\_protein | BGC0001281 | Ustilagic\_acid\_biosynthetic\_gene\_cluster | 41 | 101.78041543 | 232 | 1e-60 |
| XP\_011392733.1 | Ustilagic\_Acid\_hydroxylase | BGC0001281 | Ustilagic\_acid\_biosynthetic\_gene\_cluster | 30 | 77.1513353116 | 103 | 5.4e-22 |
| PSSDR1 |  | BGC0001325 | Noscapine\_biosynthetic\_gene\_cluster. | 30 | 85.4599406528 | 89 | 1.1e-17 |
| ABF89140.1 | NAD\_dependent\_epimerase/dehydratase\_family\_protein | BGC0000871 | VEPE\_/\_AEPE\_/\_TG-1\_biosynthetic\_gene\_cluster | 28 | 88.1305637982 | 74 | 3.5e-13 |
| AAD13546.1 | NDP-hexose\_4,6-dehydratase\_homolog | BGC0000239 | Landomycin\_biosynthetic\_gene\_cluster | 33 | 40.3560830861 | 54 | 2.9e-07 |
